# Haematinic Deficiency and Macrocytosis in Middle-Aged and Older Adults

**DOI:** 10.1371/journal.pone.0077743

**Published:** 2013-11-07

**Authors:** Therese McNamee, Trish Hyland, Janas Harrington, Sharon Cadogan, Bahman Honari, Kanthi Perera, Anthony P. Fitzgerald, Ivan J. Perry, Mary R. Cahill

**Affiliations:** 1 Department of Epidemiology & Public Health, University College Cork (UCC), Cork, Ireland; 2 Department of Haematology, Cork University Hospital (CUH), Cork, Ireland; 3 Centre for Support and Training in Analysis and Research (CSTAR), University College Dublin, Dublin, Ireland; 4 Department of Haematology, Tullamore General Hospital, Tullamore, Ireland; University of Florida, United States of America

## Abstract

**Objective:**

To assess the prevalence and determinants of haematinic deficiency (lack of B12 folate or iron) and macrocytosis in blood from a national population-based study of middle-aged and older adults.

**Methods:**

A cross-sectional study involving 1,207 adults aged ≥45 years, recruited from a sub-study of the Irish National Survey of Lifestyle Attitudes and Nutrition (SLÁN 2007). Participants completed a health and lifestyle questionnaire and a standard food frequency questionnaire. Non-fasting blood samples were obtained for measurement of full blood count and expert morphological assessment, serum ferritin, soluble transferrin receptor assay (sTfR), B12, folate and coeliac antibodies. Blood samples were also assayed for thyroid function (T4, TSH), liver function, aminotransferase (AST) and gamma-glutamyl transferase (GGT).

**Results:**

The overall prevalence (95% C.I.) of anaemia (Hb <13.5g/dl men and 11.3 g/dl women) was 4.6% (2.9%–6.4%) in men and 1.0% (0.2%–1.9%) in women. Iron deficiency (ferritin <17ng/ml men and <11ng/ml in women) was detected in 6.3% of participants (3.7% in males and 8.7% in females, p<0.001). Based on both low ferritin and raised sTfR (>21nmol/ml) only 2.3% were iron-deficient. 3.0% and 2.7% were found to have low levels of serum folate (<2.3ng/ml) and serum B12 (<120ng/l) respectively. Clinically significant macrocytosis (MCV>99fl) was detected in 8.4% of subjects. Strong, significant and independent associations with macrocytosis were observed for lower social status, current smoking status, moderate to heavy alcohol intake, elevated GGT levels, deficiency of folate and vitamin B12, hypothyroidism and coeliac disease. The population attributable fraction (PAF) for macrocytosis associated with elevated GGT (25.0%) and smoking (24.6%) was higher than for excess alcohol intake (6.3%), folate deficiency (10.5%) or vitamin B12 (3.4%).

**Conclusions:**

Haematinic deficiency and macrocytosis are common in middle-aged/older adults in Ireland. Macrocytosis is more likely to be attributable to an elevated GGT and smoking than vitamin B12 or folate deficiency.

## Introduction

Deficiency of a number of vitamins and minerals required for normal erythropoiesis (haematinics) is associated with anaemia. Deficiency of iron, B12 and folate (the most common haematinics) are the most prevalent forms of vitamin deficiency worldwide [1], [2], [3]. Individually and collectively, deficiency of iron, B12 and folate are significant causes of morbidity in the population. The estimated prevalence of haematinic deficiency varies widely with studies from Europe ranging from 5% to 46% [4],[5],[6]. In a recent Canadian population study the estimated prevalence of folate deficiency was close to zero (following flour supplementation) while B12 deficiency was estimated at approximately 5% of the population [7]. By contrast, in the NHANES 2005–2006 study in the US the prevalence of folate deficiency was estimated at 4.5% using a more sensitive measure of red cell folate [8]. 

While methods for the measurements of B12 and folate deficiency are reasonably well defined, the measurement of iron deficiency is more complex. Diagnosis of iron deficiency based on ferritin (the current standard test) may be enhanced by the addition of serum soluble transferrin receptor (sTfR) assay. Soluble transferrin receptor (sTfR), a transmembrane protein expressed abundantly on erythroid precursors in the bone marrow [9], is of value in identifying iron deficiency [10],[11],[12]. When iron stores are depleted, sTfR levels rise indicating iron-deficient erythropoiesis which precedes the development of anaemia and in some instances precedes the decline in serum ferritin concentrations [13]. Thus sTfR has the potential to identify latent iron deficiency. However, the sTfR assay is relatively expensive and is not widely used. 

Anaemia is associated with a number of classic changes in red cell morphology, depending on the underlying cause. Iron deficiency is associated with small red cells (microcytosis) whereas deficiency of B12 or folate is associated with increased red cell size (macrocytosis), expressed as mean cell volume (MCV). Laboratory evidence of microcytosis and macrocytosis and/ or iron, B12 and folate deficiency requires further assessment (including expert morphological assessment of a peripheral blood smear) to investigate a wide range of potential underlying causes. These causes include nutritional deficiency, malabsorption, blood loss, occult malignancy, excess alcohol intake, liver disease, hypothyroidism, hereditary causes of anaemia and rare bone marrow disorders such as myelodysplasia. 

While data on the prevalence of haematic deficiency and associated red cell morphological abnormalities are well documented in general population samples worldwide, data on the prevalence and relative importance of underlying conditions are relatively sparse. Data from the Republic of Ireland are needed to inform national policy on nutrition and health including the issue of folate supplementation of flour. There is a dearth of population-based data on the prevalence and determinants of macrocytosis -a frequent finding in clinical practice and associated with significant resource use in follow-up investigations. 

The aim of this study was to estimate the prevalence and major determinants of haematinic deficiency and macrocytosis in linked, anonymised blood samples drawn from a national population-based study of men and women aged 45 years and older, including expert morphological assessment of red cell morphology and assessment of a wide range of potential underlying causes.

## Methods

### Design, Sampling and Data

We have carried out a cross sectional study involving a sample of 1,207 adults aged 45 years and older, participants in the physical examination sub-study component of the third national Survey of Lifestyle, Attitudes and Nutrition in Ireland conducted in 2007 (SLÁN 2007),[14-16]. The SLÁN 2007 study is based on a nationally representative sample of 10,364 adults. The population for the survey was defined as adults aged18 years and over living in residential households in Ireland (residents of institutions, nursing homes, hospitals, prisons and homeless hostels were not included). The sampling frame used for the survey was the Geo Directory, a list of all addresses in the Republic of Ireland, which distinguishes between residential and commercial establishments. The sample was a multi-stage probability sample, where each dwelling has a known probability of selection. The overall response rate for the main survey was 62% of whom 66% consented to the physical examination sub-study. All respondents aged 45 and over who took part in the main survey were invited to take part in a physical examination sub study which was carried out at a separate time from the core survey, usually in an occupational health setting. Appointments were scheduled to suit respondents, who were all well on the day of sampling. Subjects who were unexpectedly unwell were excluded. Of the 1207 participants who agreed to take part in this sub study 67% were adults aged 45 to 64 years and 33% were adults aged 65 years and older.

Participants completed a health and lifestyle questionnaire which addressed core socio-demographic and behavioural factors including smoking and alcohol consumption using standard questionnaire instruments and a standard food frequency questionnaire (FFQ). Social class was coded using the European Socio-Economic Classification (ESeC) with levels 1-6 characterised as lower and levels 7-10 as higher [17]. A smoker was defined as a current smoker, who has smoked “at least 100 cigarettes during my lifetime” and participants were classified as either current smokers or non-smokers. Average alcohol consumption was estimated as the units of alcohol consumed per week with participants consuming 0-14 units for women and 0-21 units for men classified as within the recommended weekly limit and those consuming > 14 units for women and > 21 units for men classified as above the recommended weekly limit. The FFQ was an adapted version of the EPIC study, validated for use in the Irish population. Full details of the FFQ have been documented elsewhere [18]. Subjects were asked to indicate their average use of food items over the last year. Frequency of consumption of a medium serving or common household unit was asked for each food and later converted into quantities using standard portion sizes. The frequency categories were ‘never or less than once a month’, ‘1-3 times per month’ ‘once a week’, ‘2-4 per week’, ‘5-6 per week’, ‘once a day’, ‘2-3 per day’, ‘4-5 per day’ and ‘6+per day’. Individual food items were combined into food groups with like-constituent foods being grouped together.

### FFQ Analysis

The food frequency data was analysed using a specially designed programme FFQ Software Version 1.0, developed by Juzer Lotya, NNSC, School of Public Health and Population Science, University College Dublin. The programme converts the dietary information provided to food quantities and subsequently to food nutrient values, based on the Food Standards Agency McCance and Widdowson’s Food Composition Tables (1997).

### Blood Samples

Non-fasting blood samples were obtained for measurement of full blood count (FBC), serum ferritin, serum soluble transferrin receptor assay (sTfR), serum B12, serum folate and coeliac antibodies. Blood samples were also assayed for thyroid function (T4 and TSH) and gamma-glutamyl transferase (GGT), with the latter used as a marker of liver function. Blood film preparation and FBC analysis was carried out in Biominis (formerly Claymon) Laboratories (Sandyford, Dublin) using the Bayer Advia 120 (Dade Behring, Germany) within 48 hours after the samples were taken. The Biominis laboratories haemoglobin reference range given for males is 13.5-17.2 g/dl and females 11.3-15.2 g/dl). A total of 922 blood films were prepared by Claymon laboratories at the time of full blood count analysis.

### Morphological Analyses

Blood smear preparation was performed by Biomnis Laboratories. The blood smears were stained with May-Grunewald-Giemsa on the Quickslide Plus staining machine (GG & B Company, Texas). Each smear was assessed for 19 morphological features (including early features of haematinic deficiency, myelodysplastic syndrome (MDS), or other haematological abnormalities). All films were examined by one expert reviewer (MRC) and a sub-sample were reviewed by a blinded second expert (KP). EDTA change was assessed and graded (1-3) by each observer who also assessed and scored for the 19 pre-agreed morphological abnormalities [19].

### Serum Assays

The serum samples used had been separated after collection and stored frozen at -80°C using standard methods. Automated immunoassay analyses for B12 and folate were performed on the Beckman Coulter Unicel DXI 800 system which uses chemiluminescent technology with competitive binding immuno-enzymatic assays. A total of 1207 samples were available for analysis at the CPA accredited Cork University Hospital haematology laboratory. Ferritin levels were determined using a two-site immuno-enzymatic sandwich assay. The reference ranges from Cork University Hospital haematology laboratory for these assays are as follows; serum Folate; 2.3 – 20 ng/ml, serum B12; 120-650 ng/l and serum Ferritin; 17-320ng/ml (male) and 11-320 ng/ml (female). For serum B12, levels of 120 to 135 are reported as indeterminate to allow for the assay difficulties [20]. Due to the lack of a universal WHO standard, B12 analysis is associated with poor reproducibility between laboratories and an indeterminate range is therefore required.

Serological levels of sTfR were also measured using a chemiluminescent reaction between the substrate and the bound alkaline phosphastase conjugated anti-sTfR antibody (Beckmann Coulter). The reference range quoted by the manufacturer for sTfR is 12.16 – 27.25 nmol/L. The diagnostic cut-off recommended by the manufacturer is 21nmol/L.

Thyroid function, aminotransferase AST and Gamma-glutamyl transferase (GGT)) were analysed in Biominis Laboratories using standard automated immunoassays.

### Serological analysis for Coeliac antibodies

Coeliac antibodies were analysed in Cork University Hospital. A total of 970 samples were assayed, using two different serological assays in standard automated immunoassay. All samples were initially tested for the presence of IgA anti-tissue transglutaminase (tTG) antibodies and each positive result was then confirmed with the detection of anti-endomysial (EMA) antibodies. Those samples that tested positive for anti-tTG levels but were subsequently negative for anti-EMA were classified as negative.

### Ethical Approval

The SLÁN 2007 survey was granted ethical approval by the Research Ethics Committee of the Royal College of Surgeons in Ireland (RCSI). This project required additional laboratory work at Cork University Hospital on stored blood samples that were linked to the original data set but fully and irreversibly anonymised in compliance with a protocol approved by the SLÁN 2007 national steering group. The current research based on these samples was approved by the Cork Teaching Hospitals Research Ethics Committee. 

### Statistical Analyses

Data merging and analyses were performed using Stata™ V. 12.1. The core sample was weighted to closely approximate the Census 2006 figures for gender, age, marital status, education, occupation, region, household size and ethnicity. As there was a tendency for those with lower levels of education and in lower socio-economic classes to be under-represented in the physical examination sub-study, the physical examination data were re-weighted to reflect the Republic of Ireland’s population age 45 years and above. The weights were assigned to each case in the data file using the pweights option in Stata.

 Chi-squared tests and T-tests were used to compare proportions and means for categorical and continuous variables respectively. Factors associated with macrocytosis were studied using multiple logistic regression analyses. As the analyses presented are based on a cross-sectional study, the prevalence odds ratio has been used to approximate relative risks. In these analyses, macrocytosis ((MCV >99fl: the outcome variable) was fitted as a categorical variable. Similarly, in these analyses, the explanatory variables (age group, gender, social class and education, alcohol consumption, smoking status, coeliac disease status and serum levels of GGT, TSH, Folate, B12) were fitted as categorical variables using standard, clinically appropriate cut-points. Prevalence odds ratios from univariate analyses and following adjustment for age and gender are presented with 95% Confidence Intervals and p values estimated from the regression coefficients in the multiple logistic regression analyses.

The population attributable fraction (PAF) for specific modifiable factors associated with macrocytosis was estimated as follows: PAF = Pe (RR-1)/ Pe (RR-1) +1 where Pe is the proportion of the source population exposed to the risk factor and RR refers to the magnitude of the relative risk of macrocytosis associated with the specific exposure [21]. Of the 1,207 samples analysed, data on sample weights and other variables were not available for a total of eight individuals. Thus all analyses presented are based on a sample of 1199. 

The SLAN main survey data is publically archived in the Irish Social Science Data Archive and is accessible through  http://www.ucd.ie/issda/.  The physical examination data, including the data from the additional analyses of the stored blood analysis will be archived November/December 2013.

## Results

Table 1 shows the breakdown of core socio-demographic characteristics of the study sample and relevant clinical variables by gender. The men were younger and there were significant differences in social class, educational attainment, smoking status and alcohol intake by gender. The data showed that 31.3% of men and 13.2% of women had evidence of abnormal liver function based on elevated gamma glutamyl transferase (GGT) concentrations (GGT> 48 u/L). A total of 3.9% of the sample had TSH levels consistent with hypothroidism and 0.9% of the sample tested positive for coeliac disease, (Table 1). 

**Table 1:** Socio-Demographic Characteristics of the Study Sample and Relevant Clinical Variables by Gender.

**Variable**	**All (N=1191)**	**Men (N=519)**	**Women (N=672)**	
	**N (%)**	**N (%)**	**N (%)**	P-value*
**Age**				< 0.001
45-64yrs	798 (67.0%)	361(69.6%)	434 (64.6%)	
65+yrs	393 (33.0%)	158 (30.4%)	238 (35.4%)	
**Social Class** ^a^				< 0.001
Upper	790 (66.3%)	386 (74.3%)	395 (58.8%)	
Lower	401 (33.7%)	133 (25.7%)	277 (41.2%)	
**Education**				< 0.001
Primary	456 (38.3%)	206 (39.7%)	249 (37.0%)	
Secondary	494 (41.5%)	200 (38.6%)	297 (44.2%)	
Tertiary	241 (20.2%)	113 (21.7%)	126 (18.8%)	
**Smoking**				< 0.001
Non-smoker	958 (80.4%)	421 (81.%)	536 (79.8%)	
Smoker	233 (19.6%)	98 (18.9%)	136 (20.2%)	
**Alcohol** ^b^				< 0.001
Within Rec limit	1085 (91.1%)	454 (87.4%)	640 (95.2%)	
Above Rec limit	106 (8.9%)	64 (12.6%)	32 (4.8%)	
**Clinical Variables**				
Elevated GGT^c^	245 (21.1%)	160 (31.5%)	85 (13.2 %)	< 0.001
Hypothyroid^d^	47 (3.9%)	12 (2.7%)	35 (5.1%)	0.04
Coeliac disease	11 (0.9%)	5 (1.1%)	6 (0.7%)	0.46

a Social Class: Upper = Level 1-6, Lower = Level 7-10

b Alcohol: Within Recommended limit = <14 units per week for women & <21 units per week for men, Above Recommended limit = >14 units per week for women & >21 units per week for men.

c Elevated GGT: GGT> 48 IU/L

d Hypothyroid: TSH>4.2 mIU/L

* Chi-squared test

Table 2 shows estimated dietary intakes of iron, folic acid and vitamin B12 by gender and age group. Iron intakes were higher in men than in women and a substantial proportion of the population sample had estimated iron intakes below the Recommended Daily Allowance (RDA) ranging from 31% in men aged 45-64 years to 70% in women in the 45-64 age group. Small but statistically significant gender differences in estimated dietary folic acid and vitamin B12 intakes were also observed (Table 2). 

**Table 2:** Estimated Dietary Intakes of Iron, Folic Acid and Vitamin B12 by Gender and Age Group.

**FFQ-estimated dietary intake**		**Age Group**	**All (N=1191)**			**Men (N=519)**			**Women (N=672)**			P-value*
			**Mean (SD**)	**% below RDA**		**Mean (SD**)	**% below RDA**		**Mean (SD**)	**% below RDA**		
Dietary Iron mg/d		45-64 ^a^	12.6 (5.4)	50.9		12.8 (5.8)	31.4		12.4 (5.1)	70.1		<0.001
		65+ ^b^	13.4 (7.7)	31.7		14.4 (9.6)	33.9		12.6 (5.7)	29.6		<0.001
Dietary Folate µg/d^c^		45-64 ^c^	337.5 (146.0)	13.8		331.9 (156.2)	14.4		343.1(135.0)	13.3		<0.001
		65+ ^c^	346.7 (185.3)	10.4		358.6 (237.1)	12.6		337.8(132.4)	8.7		<0.001
Dietary Vit B12 µg/d^d^		45-64 ^d^	5.2 (3.7)	18.3		5.1 (3.6)	17.8		5.3 (3.8)	18.8		<0.001
		65+ ^d^	5.4 (5.4)	18.7		5.7 (5.7)	21.1		5.2 ( 5.1)	17.0		<0.001

a Dietary Iron RDA Age 45-64 years: Men: 10mgs/d and Women: 14mgs/d

b Dietary Iron RDA Age 65+ years: Men: 10mgs/d and Women: 9mgs/d

c Dietary Folate RDA Age 45-64 years and 65+ years: 200 µg/d

d Dietary Vit B12 RDA Age 45-64 years and 65+ yea**rs**: 2.5 µg/d

* T-test and Chi-squared tests for continuous and categorical variables respectively

**Figure 1:**
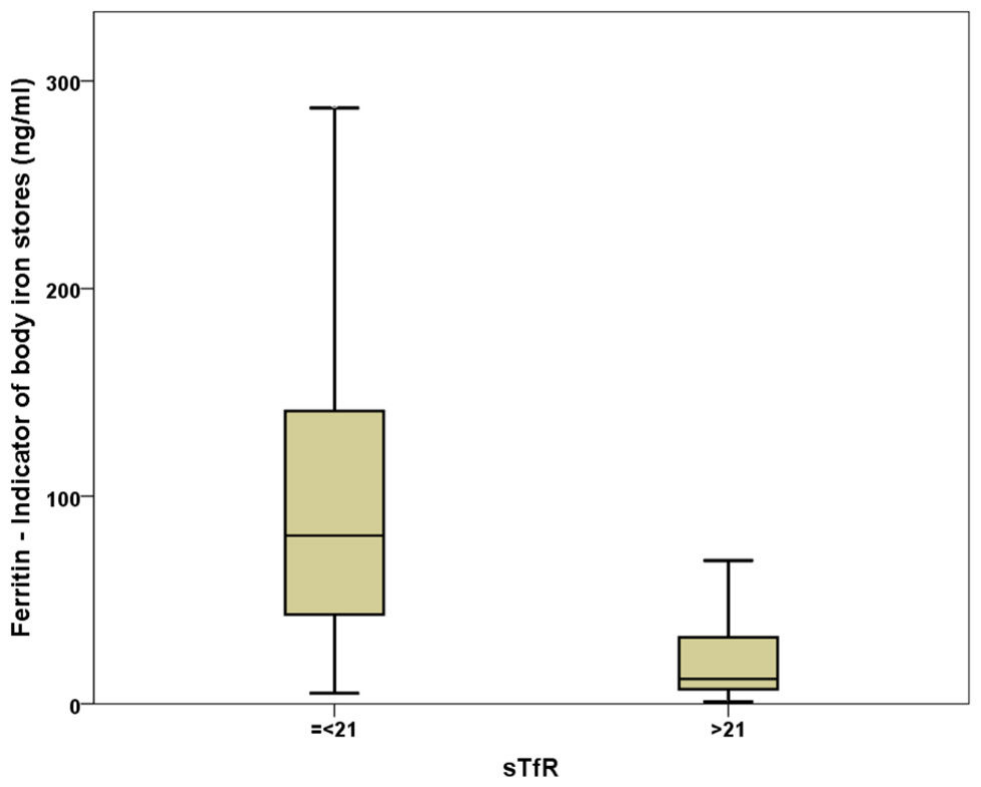
Distribution of ferritin in subjects with normal sTfR (=<21nmol/L) and those with elevated sTfR (>21nmol/L).

Mean haemoglobin concentrations were higher in men than in women. However the prevalence of anaemia was also higher in men (4.6%) than in women (1.0%) reflecting the different cut-off points for anaemia in men and women, (Table 3). As expected, the prevalence of iron deficiency based on low serum ferritin was considerably higher in women (8.7%) than in men (3.7%). Similarly, the prevalence of raised sTfR (>21nmol/ml) consistent with iron deficiency was higher in women (7%) than in men 3.2%. In men with estimated iron intakes below the RDA, 68.4% had low serum ferritin and 80.8% had an elevated sTfR. Similarly, 63.1% of women with estimated iron intakes below the RDA had low serum ferritin and 67.9% had an elevated sTfR. Based on both low ferritin (<17ng/ml for men and <11ng/ml for women) and raised sTfR, 2.3% (0.9% men and 3.8% women) were iron-deficient on both criteria, 3.9% (2.8% men and 5.0% women) were iron-deficient by ferritin but not sTfR and 2.8% (2.3% men and 3.2% women) were classified as deficient by sTfR but not ferritin (latent iron deficiency) –see Figure 1. The estimated prevalence of iron deficiency defined on the basis of either low ferritin or raised sTfR was 9%. 

**Table 3:** Haematinic Deficiency and Relevant Covariates by Gender in Middle-Aged and Older Irish Adults.*

**Variable**	**All (*n*=1191)**	**Men (*n*=519)**	**Women (*n*=672)**	**P-Value** **
**Hb (g/dl)**				
Mean (SD)	14.53 (1.28)	15.25 (1.15)	13.86 (0.99)	< 0.001
% below cut off^a^, 95% CI	2.8 (1.8-3.7)	4.6 (2.9-6.4)	1.0 (0.2-1.9)	
**Anaemia**				
%, 95% CI	2.8 (1.8-3.7)	4.6 (2.9-6.4)	1.0 (0.2-1.9)	< 0.001
**MCV (fl)**				
Mean (SD)	92.3 (5.0)	92.6 (5.4)	92.0 (4.7)	
% below cut off^b^, 95% CI	0.50 (0.1-0.9)	0.60 (0.00-1.2)	0.5 (0.0-1.0)	0.82
% above cut off, 95% CI	8.4 (6.8-10.0)	9.1 (6.7-11.5)	7.9 (5.7-10.1)	0.46
**Ferritin (ng/ml)**				
Mean (SD)	107.1 (112.2)	144.6 (134.6)	71.6 (69.1)	
% below cut off^c^, 95% CI	6.3 (4.9-7.7)	3.7 (2.1-5.3)	8.7 (6.4-11.0)	< 0.001
**sTfR (nmol/ml)**				
Mean (SD)	14.7 (4.2)	14.7 (3.6)	14.7 (4.7)	
% above cut off^d^, 95% CI	5.1 (3.9-6.3)	3.2 (1.7-4.7)	7 (5.1, 8.9)	0.004
**Serum Folate (ng/ml)**				
Mean (SD)	8.3 (4.7)	8.2 (4.6)	8.4 (4.8)	
% below cut off^e^, 95% CI	3.0 (2.0-4.0)	3.7 (2.1-5.3)	2.5 (1.2-3.8)	0.23
**Serum Vit B_12_ (ng/l)**				
Mean (SD)	293.5 (136.1)	276.4 (99.9)	309.5 (161.5)	
% below cut off^f^, 95% CI	2.7 (1.7-3.7)	1.3 (0.3-2.3)	4.0 (2.4, 5.6)	0.005

* The numbers may vary for specific variables due to missing values

a Hb lower cut-offs: <13.5g/dl men and <11.3 g/dl women

b MCV lower cut-off: <80fl and upper cut-off: >99fl

c Ferritin lower cut-offs: <17ng/ml men and <11ng/ml in women

d sTfR above cut-off: (>21nmol/ml)

e Serum Folate lower cut-off: <2.3ng/ml

f Serum Vit B_12_ lower cut-off: <120ng/l

** T-test and Chi-squared tests for continuous and categorical variables respectively

Overall, 3.0% and 2.7% of the sample had low serum folate (<2.3ng/ml) and serum B12 (<120ng/ml) concentrations respectively with no significant gender differences in folate concentrations but a higher prevalence of Vitamin B12 deficiency in women (Table 3). Mean serum folate in those with folate intakes below the RDA was 2.0ugm/day versus 8.6ugm/day in those with estimated intakes above the RDA. Mean serum B12 in those with estimated dietary B12 intakes below the RDA was 99.0ug/day versus 298.8ug/day in those with estimated intakes above the RDA. Subjects aged 65 or over had a significantly higher prevalence of Vitamin B12 deficiency compared to subjects aged 45-64 (5.0% vs. 1.6% respectively, p=0.01). On cross tabulation of the serum folate and serum Vitamin B12 data, only two subjects had both low B12 (<120ng/l) and folate (<2.3ng/ml) concentrations. 

Clinically significant macrocytosis (MCV>99fl) was detected in 8.4% of participants whereas microcytosis was uncommon, Table 3. No evidence of myelodysplasia or other bone marrow disorders were detected from morphological assessment of blood films. Macrocytosis was more common in men and in younger participants. In univariate and multivariate analyses,(Table 4) strong and highly significant associations with macrocytosis were observed for lower social status, current smoking status and alcohol intake above the recommended limits, elevated gamma glutamyl transferase (GGT) levels, deficiency of folic acid and vitamin B12, hypothyroidism and coeliac disease. The association between smoking and macrocytosis was not attenuated on adjustment for alcohol and GGT. The population attributable fractions (PAF) for major factors associated with macrocytosis were as follows: alcohol intake above recommended limit 6.3% (95% CI: 4.3% - 7.6%), elevated GGT 25.0% (95% C.I: 22.5% - 27.5%), current smoking status 24.6% (95% CI: 22.1% - 27.0%), deficiency of folic acid 10.5% (95% C.I: 8.8% - 12.3%) and vitamin B12 deficiency 3.4% ( 95% C.I: 2.4% - 4.5%). 

**Table 4:** Univariate and Multivariate Associations with Macrocytosis > 99 fl.

	**Univariate Model**			**Age & Gender Adjusted Model**		
**Variable**	**OR (95% CI)**	**P-value**		**OR (95% CI)**	**P-value**	
**Age (ref: 45-64)**						
65+	0.90 (0.85, 0.95)	<0.001		-	-	
**Gender (ref: Male)**						
Female	0.48 (0.45, 0.50)	<0.001		-	-	
**Social class** (**ref: Lower**)^a^						
Higher	0.38 (0.36, 0.41)	<0.001		0.34 (0.32, 0.36)	<0.001	
**Education (ref: Primary)**						
Secondary	0.77 ( 0.72, 0.81)	<0.001		0.75 (0.71, 0.80)	<0.001	
Tertiary	0.20 (0.18, 0.22)	<0.001		0.19 (0.17, 0.21)	<0.001	
**Alcohol** (**ref**: Within Rec limit) ^b^						
Above Rec limit	1.72 (1.57, 1.88)	<0.001		1.41 (1.28, 1.54)	<0.001	
**Smoking (ref: Non-Smoker)**						
Smoker	2.89 (2.72, 3.06)	<0.001		2.91 (2.75, 3.09)	<0.001	
**GGT IU/L (ref: <48)**						
>=48	2.65 (2.50, 2.81)	<0.001		2.26 (2.13, 2.40)	<0.001	
**TSH mIU/L** (**ref: <4.2**) ^c^						
>=4.2	4.81 (3.57, 6.49)	<0.001		4.05 (3.00, 5.47)	<0.001	
**Folate ng/ml (ref: >2.3)**						
=<2.3	6.90 (6.31, 7.55)	<0.001		6.74 (6.15, 7.39)	<0.001	
**B12 ng/l (ref: >120)**						
=<120	2.48 (2.19, 2.79)	<0.001		3.40 (3.0, 3.85)	<0.001	
**Coeliac Disease (ref: Negative)**						
Positive	4.62 (3.89, 5.48)	<0.001		4.66 (3.92, 5.54)	<0.001	

a Social Class: Upper = Level 1-6. Lower = Level 7-10

b Alcohol: Within Recommended limit = <14 units per week for women & <21 units per week for men, Above Recommended limit = >14 units per week for women & >21 units per week for men.

c TSH below the lower cut-off of <0.38 were excluded from this analysis

## Discussion

This study provides data on the prevalence of haematinic deficiency and macrocytosis and the relative importance of major underlying causes of relevance to both clinical practice and public health nutrition policy. Based on the laboratory reference ranges for haemoglobin, the estimated prevalence of anaemia was higher in men (4.6%) than in women (1%). However the prevalence of iron deficiency was as expected substantially higher in women than in men. The findings highlight the importance of nutritional deficiency as a potential cause of iron, B12 and folate deficiency in a developed country setting. Consistent with previous work, coeliac disease is not an important contributor to the burden of iron deficiency in Ireland [22]. In the context of iron deficiency, the findings also highlight the impact of using the soluble transferrin receptor assay in addition to serum ferritin on the estimated prevalence of iron deficiency. The findings confirm the role of excess alcohol intake, abnormal liver function, hypothyroidism and coeliac disease as determinants of macrocytosis in addition to serum B12 and folic acid deficiency. However the findings on smoking and macrocytosis were unexpected. In this study smoking was on a par with abnormal liver function as a contributor to the population prevalence of macrocytosis, accounting for approximately 25% of cases, substantially higher than excess alcohol intake (6.3%), deficiency of folic acid (10.5%) or vitamin B12 (3.4%.).

The prevalence of anaemia is high in Ireland, especially in men. In Ireland laboratories use different lower cut off points below which anaemia is defined –varying from 13-14gm/dl in men and 11.5-12.5gm/dl in women. Based on both low ferritin and raised soluble transferrin receptor assay (sTfR), 2.3% of the sample were iron-deficient on both criteria whereas 9.0% could be classified as iron-deficient on the basis of either low ferritin or raised sTfR. Thus it is clear that the addition of sTfR to current clinical screening protocols would substantially increase the prevalence of diagnosed iron deficiency. However, if sTfR was added to serum ferritin as a confirmatory test for iron deficiency prior to referral for specialist investigation (such as endoscopy) the number investigations would decrease with potential reductions in patient morbidity and cost to health services. The sTfR assay is an expensive test and not as well validated in large population samples as ferritin [12]. Nonetheless, if the sTfR assay was introduced into routine clinical practice it would be widely requested as suspected iron deficiency is common. However, if the test was introduced in parallel with a reduction in the lower limit of haemoglobin ranges and a sex specific normal range for ferritin, it would add a margin of safety to ensure that genuinely latent iron deficiency was not ignored – an important issue in the elderly where cancer becomes a more common cause of anaemia. Because ferritin levels rise as part of the acute phase response, with fatty liver and with increased alcohol consumption, this masks co-existing iron deficiency, further highlighting the potential utility of a sTfR. Our data suggest that further analysis of the use, economic benefit and impact on clinical outcomes of the sTfR assay is warranted.

Numerous studies have demonstrated that vitamin B12 deficiency increases with age, [23],[24], with reports of between 10% to 15% of people aged over 60 years with low vitamin B12 levels [25]. By comparison the prevalence of B12 deficiency in this Irish population sample is low at 2.7%. Levels Vitamin B12 of between 120 to 135 ng/l which are reported as indeterminate in the participating laboratory were classified as normal in this study. However, only 1.6% of participants had vitamin B12 concentrations within this indeterminate range. Although published data suggests that the age related decrease in B12 levels may be attributed to poor intake or increased losses [24], estimated mean dietary intakes in both middle-aged and older participants in this study were over 5.0 µg/day which is more than twice the recommended daily intake of 2.4ug/day for men and women [26]. Thus it is likely that the low prevalence of B12 deficiency observed in this study reflects relatively high intakes of cobalamin in the middle-aged and older Irish population.

Low folate levels were also uncommon among the Irish population. However, in contrast to vitamin B12, this estimate concurs with other internationally published data on the prevalence of folate deficiency [27],[24]. The estimated mean daily intake of folic acid amongst this Irish cohort was 339µg. This is well above the recommended daily allowance of 200ug/day [26], for male and females (excluding peri-conceptional females). This study found that folate levels did not increase with age such that only 2.1% of 65 years and over were found to be folate-deficient in contrast to 3.5% with low folate levels in the 45-64 age group. In Ireland, folic acid fortification is voluntary but widespread. One of the concerns surrounding mandatory folate fortification is the potential masking of B12 deficiency in those afflicted with cobalamin deficiency [6]. This is unlikely to be a significant issue in Ireland given the current findings on vitamin B12 intakes and the low prevalence of B12 deficiency. Concern has also been raised regarding the role of unmetabolized folic acid with the possibility that excess levels of oxidised unmetabolised folate could promote cancer growth [28]. The assay used in this study detects all forms of folate including the unmetabolised fractions and 0% of participants had folate levels above the current reference range of 20ng/ml. 

The estimated prevalence of macrocytosis at 8.4% is higher than previously reported estimates of between 1.7% and 3.6% [29]. Of those participants found to be B12 and folate-deficient an extremely small proportion (0.1%) had a raised MCV, a finding which is significantly lower than reports from other groups [30]. While the association between smoking and macrocytosis is under-appreciated it has been previously described. In a paper from Meade’s group published in 1978 a small independent effect of smoking, alcohol and the oral contraceptive pill on mean cell volume (MCV) was noted [31]. Subsequently, Eschwege et al in 1979 described a more marked elevation of MCV in patients with alcoholic liver disease who were also smokers [32]. A later study describes small, but significant increases in MCV with smoking [33]. In the current study the risk of macrocytosis was increased approximately three-fold in smokers relative to non smokers. It may be objected that this association is confounded by alcohol intake. However in further multivariate analyses (not shown) the association between smoking and macrocytosis was essentially unchanged (marginally stronger) on adjustment for age, gender, alcohol intake and GGT, with the latter fitted as a continuous variable to minimise residual confounding. A possible effect of smoking on aminolevulinic acid synnthetase has been postulated little research has been done in this area [34].

This study has significant strengths and some important limitations which should be noted. The study sample is nationally representative and data are available on a wide range of relevant nutritional, lifestyle, socio-demographic and clinical variables which have allowed for a relatively detailed and comprehensive analyses of the determinants of haematinic deficiency and macrocytosis. The findings provide important reference data for other studies drawing on general population samples. A particular strength of our study is the availability of morphological analysis of the blood smears by expert reviewers, allowing us to exclude significant evidence of haemolytic anaemia and thalassaemia (conditions associated with enhanced erythropoietic activity) and increased sTfR levels not associated with iron deficiency. However, the sample size is relatively small for a national survey and no data on young adults were available. In addition, data on clinical follow-up for occult malignancy are lacking and we do not have data on inflammatory markers (C-reactive protein or erythrocyte sedimentation rate), which are important in the interpretation of serum ferritin concentrations. Mild EDTA change was widespread on the films examined. However, this mirrors the situation in most haematology laboratories which accept samples referred from community settings.

## Summary

We have described the prevalence and major nutritional, lifestyle and clinical determinants of haematinic deficiency and macrocytosis in a representative sample of Irish middle-aged men and women. The use of the soluble transferrin receptor assay has considerable potential in the routine assessment of iron deficiency and there is a need for additional studies addressing the overall cost effectiveness of this assay in different clinical settings. The findings on macrocytosis, if replicated in further cross-sectional and cohort studies, suggest that smoking should be added to the list of major causes of this common haematological abnormality.
